# Supplementary material for: Polyunsaturated Fatty Acid-Enriched Lipid Fingerprint of Glioblastoma Proliferative Regions Is Differentially Regulated According to Glioblastoma Molecular Subtype
Source: Int J Mol Sci. 2022 Mar 9;23(6):2949. doi: 10.3390/ijms23062949 (PMC8949316; doi:10.3390/ijms23062949)

Supplementary Table S1. GBM lipidomic data expressed in mean ± SEM, of the total lipid class % for each sample type.

	Healthy DMSO		Healthy TMZ		Tumor DMSO		Tumor TMZ	
	Mean	SD	Mean	SD	Mean	SD	Mean	SD
**PE plasmalogens**								
**PE P-34:0**	**ND**	-	**ND**	-	**1.6**	0.9 [aaa.b]	**0.9**	0.4 [aaa.b]
**PE P-34:1**	**13.7**	5.7	**5.0**	2.7	**8.5**	5.4	**10.3**	4.6
**PE P-34:2**	**5.0**	3.2	**ND**	-	**1.3**	1.9	**1.3**	1.1
**PE P-36:0**	**ND**	-	**ND**	-	**0.9**	0.2 [a,b]	**0.3**	0.0
**PE P-36:1**	**8.6**	-	**2.8**	1.1	**4.3**	1.4	**3.7**	1.0
**PE P-36:2**	**20.8**	7.1	**7.3**	3.9 [aaa]	**3.6**	2.1 [aaa]	**3.6**	1.5 [aaa]
**PE P-36:3**	**1.7**	0.9	**0.9**	-	**1.6**	0.7	**1.9**	0.5
**PE P-36:4**	**1.9**	0.7	**3.8**	3.4	**14.8**	5.4 [aaa,bb]	**20.4**	3.6 [aaa,bbb]
**PE P-38:1**	**1.0**	0.4	**0.4**	0.3	**0.7**	0.5	**0.4**	-
**PE P-38:2**	**3.9**	-	**1.8**	-	**0.7**	0.4	**1**	0.8
**PE P-38:4**	**14.7**	2.2	**20.4**	4.5	**22**	6.4	**21.8**	3.6
**PE P-38:5**	**7.1**	0.6	**8.1**	2.5	**8.0**	3.3	**8.3**	2.0
**PE P-38:6**	**2.8**	1.5	**6.5**	1.3	**10.3**	2.7 [aaa]	**11.1**	3.4 [aaa,b]
**PE P-40:3**	**0.9**	0.1	**0.9**	0.2	**1.8**	1.5	**0.7**	0.6
**PE P-40:4**	**8.8**	3.1	**11.5**	2.9	**5.6**	2.0 [b]	**3.6**	2.3 [bb]
**PE P-40:5**	**7.1**	1.6	**6.2**	0.9	**4.3**	2.0	**3.3**	1.6 [aa]
**PE P-40:6**	**10.2**	10.8	**23.5**	8.0	**10.4**	3.6	**7.6**	2.7 [b]
**PE P-40:7**	**1.8**	0.8	**3.2**	0.1	**1.8**	1.0	**1.3**	1.1
**PE P-42:5**	**0.6**	-	**ND**	-	**0.6**	0.4	**0.4**	-
**PE P-42:6**	**ND**	-	**ND**	-	**0.7**	0.5	**0.5**	-
**PE**								
**PE 32:0**	**0.3**	0.1	**0.4**	0.1	**0.5**	0.4	**0.7**	0.3
**PE 34:0**	**3.2**	0.9	**5.5**	1.6	**5.4**	1.7	**5.1**	1.3
**PE 34:1**	**5.5**	2.1	**3.8**	1.7	**6.2**	1.9	**6.6**	4.5 [b]
**PE 34:2**	**ND**	-	**ND**	-	**1.2**	1.9	**1.4**	0.8
**PE 36:0**	**1.8**	-	**ND**	-	**2**	1.3	**2.4**	1.6
**PE 36:1**	**35.6**	10.4	**27.1**	5.2	**30**	7.9	**31.7**	7.4
**PE 36:2**	**7.4**	3.3	**3.9**	1.8	**10.1**	9.5	**13.1**	6.8
**PE 36:3**	**0.7**	-	**ND**	-	**1.5**	1.6	**2.1**	1.4
**PE 36:4**	**0.6**	0.2	**1.0**	0.3	**1.1**	0.5	**1.7**	0.3 [aa]
**PE 38:0**	**0.0**	-	**0.0**	-	**0.3**	0.1	**0.2**	0.1
**PE 38:1**	**6.1**	1.7	**4.0**	0.7	**3.9**	1.8	**2.5**	0.8 [aa]
**PE 38:2**	**4.2**	1.8	**2.3**	1.2	**5.9**	1.6 [b]	**5.5**	2.3

PE 38:3	**ND**	-	**ND**	-	**1.5**	-	**ND**	-
PE 38:4	**11.3**	3.3	**15.8**	3.3	**13.4**	4.1	**13.9**	3.1
PE 38:5	**2.6**	0.6	**2.4**	0.9	**2.3**	0.6	**4.0**	1.2 [c]
PE 38:6	**2.3**	1.9	**4.3**	0.9	**1.9**	0.9 [b]	**2.2**	0.9
PE 40:1	**ND**	-	**ND**	-	**ND**	-	**ND**	-
PE 40:3	**0.6**	-	**ND**	-	**0.2**	-	**0.5**	0.1
PE 40:4	**5.2**	2.2	**6.6**	1.8	**5.5**	3.8	**3.3**	1.1
PE 40:5	**2.3**	-	**ND**	-	**2.6**	0.5	**2.5**	0.4
PE 40:6	**13.9**	11	**23.2**	7.7	**6.2**	3.3 [b]	**4.0**	1.9 [b]
PE 40:7	**1.0**	0.3	**1.4**	0.4	**1.4**	0.6	**1.4**	0.8
**PI**								
PI 18:0	**0.5**	0.4	**ND**	-	**0.3**	0.2	**ND**	-
PI 18:1	**1.5**	2.0	**0.2**	0.2	**ND**	-	**ND**	-
PI 24:2	**2.9**	-	**ND**	-	**ND**	-	**ND**	-
PI 34:0	**ND**	-	**ND**	-	**0.3**	ND	**2.4**	3.3
PI 34:1	**13.7**	4.3	**ND**	-	**4.8**	2.7 [a]	**4.6**	4.0 [a]
PI 36:1	**4.5**	2.7	**2.1**	0.8	**2.4**	0.8	**35.7**	-
PI 36:2	**2.6**	1.2	**1.7**	-	**5.5**	5.5	**6.6**	5.5
PI 36:4	**8.3**	1.7	**10.9**	-	**5.1**	1.4	**6.5**	5.7
PI 38:2	**ND**	-	**ND**	-	**0.9**	0.4	**3.6**	0.0 [cc]
PI 38:4	**54.9**	8.4	**80.4**	7.2	**75.7**	11.1	**65**	29.4
PI 38:5	**5.1**	0.3	**6.3**	0.5	**5.5**	1.5	**5.5**	1.7
PI 38:6	**0.8**	0.5	**ND**	-	**ND**	-	**ND**	-
PI 40:0	**7.1**	-	**ND**	-	**ND**	-	**ND**	-
PI 40:1	**6.0**	4.3	**2.7**	1.4	**ND**	-	**ND**	-
PI 40:4	**1.4**	0.0	**ND**	-	**1.6**	0.2	**3**	0.3 [aa,cc]
PI 40:5	**ND**	-	**ND**	-	**1.3**	0.8	**1.5**	1.2
PI 40:6	**2.8**	1.8	**5.4**	2.1	**2.8**	1.3	**2.7**	2.4
PI 40:7	**0.1**	ND	**0.6**	0.4	**0.2**	0.1	**ND**	-
**SM**								
SM d32:2	**0.6**	0.8	**ND**	-	**1.0**	0.7	**0.5**	0.3
SM d34:0	**ND**	-	**ND**	-	**2.4**	1.1	**2.7**	1.3
SM d34:1	**10.6**	4.4	**10.7**	5.9	**37.5**	15.5 [aa,bb]	**33.1**	8.7 [aa,b]
SM d34:2	**1.0**	0.1	**1.1**	0.9	**3.9**	3.9	**2.0**	1.2
SM d36:0	**ND**	-	**ND**	-	**2.8**	1.1	**3.5**	1.1
SM d36:1	**41.0**	8.0	**50.1**	9.0	**26.6**	16.2	**34.6**	19.3
SM d36:2	**8.3**	2.8	**9.7**	3.7	**8.3**	5.2	**9.9**	3.4
SM d38:1	**6.6**	6.6	**12.4**	6.3	**1.7**	0.7 [b]	**1.7**	0.2
SM d40:1	**0.8**	0.3	**1.2**	0.5	**3.2**	1.7	**2.9**	1.6
SM d40:2	**ND**	-	**ND**	-	**1.8**	1.4	**1.3**	1.0
SM d42:1	**3.4**	1.0	**1.3**	0.5	**5.4**	3.9	**1.9**	0.8
SM d42:2	**26.8**	7.7	**12.2**	5.8	**9.4**	3.5	**14.5**	8.9
SM d42:3	**1.3**	0.4	**1.1**	0.6	**3.6**	2.0	**2.3**	2.6

**SM d44:2**	**2.9**	1.0	**1.2**	0.7	**ND**	-	**ND**	-
**Sulfatides**								
**Sulf d34:2**	**0.4**	-	**1.7**	1.1	**5.4**	4.2	**3.7**	3.3
**Sulf d34:3**	**0.3**	0.2	**1.7**	1.8	**9.6**	5.9	**11.9**	13.5
**Sulf d36:0**	**ND**	-	**ND**	-	**ND**	-	**2.5**	0.4
**Sulf d36:1**	**3.4**	0.7	**3.0**	0.8	**3.9**	4.4	**14.7**	8.1 [aa,bb,c]
**Sulf d36:2**	**0.4**	0.1	**0.7**	0.3	**1.3**	0.8	**9.3**	14.2
**Sulf d36:3**	**1.4**	1.2	**6.2**	4.3	**26.2**	17.8	**14.2**	11.3
**Sulf d36:4**	**0.2**	0.2	**0.7**	0.4	**11.4**	10.9	**10.5**	15
**Sulf d38:6**	**ND**	-	**ND**	-	**9.1**	0.0	**5.6**	1.9
**Sulf d40:1**	**1.3**	0.2	**1.0**	0.2	**0.2**	-	**2.3**	0.7 [a,bb]
**Sulf d40:6**	**0.8**	-	**1.9**	1.1	**8.0**	10.9	**0.7**	0.4
**Sulf d42:1**	**11.3**	5.7	**6.6**	2.3	**3.8**	3.5	**7.1**	5.8
**Sulf d42:2**	**50.8**	5.5	**49.4**	7.0	**29.9**	20.4	**40.6**	19.7
**Sulf d44:2**	**10**	1.2	**10.2**	1.5	**5.3**	4.1	**10.3**	1.4 [aa,bb,cc]
**Sulf d44:3**	**1.5**	0.5	**1.9**	0.2	**ND**	-	**1.5**	-
**Sulf t40:1**	**2.7**	0.6	**4.0**	1.7	**1.3**	1.5	**2.7**	-
**Sulf t40:2**	**0.3**	0.2	**0.6**	0.4	**ND**	-	**ND**	-
**Sulf t40:4**	**0.7**	-	**1.4**	0.5	**4.8**	5.0	**5.5**	7.5
**Sulf t42:1**	**15.9**	1.3	**19.6**	3.8	**9.0** [b]	5.7	**ND**	-
**Sulf t44:1**	**1.0**	0.3	**1.4**	0.3	**ND**	-	**ND**	-
**DG**								
**DG 32:0**	**2.5**	1.3	**2.0**	1.0	**7.8**	4.9	**9.5**	7.7
**DG 32:1**	**3.0**	3.0	**4.1**	3.8	**4.5**	0.9	**4.2**	3.0
**DG 32:2**	**3.3**	1.6	**3.1**	2.1	**2.8**	0.9	**4.9**	3.4
**DG 34:0**	**4.5**	1.4	**2.9**	0.7	**5.9**	1.2	**7.7**	4.7
**DG 34:2**	**58**	11.2	**51.6**	10.1	**33.3**	17.8	**41**	18.5
**DG 34:3**	**4.4**	2.5	**3.3**	1.8	**10.0**	6.9	**9.3**	6.3
**DG 36:2**	**6.1**	5.6	**7.2**	4.2	**5.2**	4.6	**5.0**	1.3
**DG 36:3**	**2.8**	2.7	**3.8**	1.1	**7.1**	1.6	**2.3**	2.6 [c]
**DG 36:4**	**1.3**	0.8	**2.1**	0.5	**2.9**	3.4	**3.9**	1.6
**DG 36:5**	**4.5**	3.3	**5.4**	3.4	**7.1**	6.0	**5.7**	4.4
**DG 38:4**	**1.0**	0.4	**1.9**	1.0	**0.8**	1.0	**0.5**	0.4
**DG 38:5**	**4.2**	3.4	**5.5**	3.4	**6.9**	5.5	**3.6**	1.3
**DG 38:6**	**1.1**	0.7	**1.7**	0.4	**2.4**	1.0	**1.6**	0.5
**DG 38:7**	**1.9**	1.8	**3.2**	1.9	**2.1**	0.7	**0.7**	0.7
**DG 40:7**	**1.2**	1.3	**2.4**	1.2	**1.2**	0.7	**0.2**	0.3 [b]
**TG**								
**TG 36:4**	**0.4**	0.1	**0.4**	0.3	**1.0**	0.4	**0.9**	0.9
**TG 38:4**	**5.1**	5.2	**10.2**	3.9	**8.0**	5.7	**9.0**	2.2
**TG 38:5**	**3.3**	2.6	**3.9**	2.0	**4.7**	1.1	**6.6**	3.1
**TG 40:5**	**55.1**	2.7	**56.1**	2.3	**42.6**	15.1	**47.4**	15.1
**TG 40:6**	**3.6**	1.7	**3.1**	1.3	**13.5**	9.6	**11**	9.1

**TG 40:7**	**1.0**	0.4	**0.9**	0.3	**1.4**	0.4	**1.7**	1.2	
**TG 42:5**	**7.7**	4.3	**8.1**	2.5	**4.8**	3.6	**2.7**	2.4	
**TG 42:6**	**4.6**	1.2	**3.4**	0.5	**8.1**	2.3 [b]	**7.0**	4.0	
**TG 42:7**	**8.8**	6.8	**3.5**	1.8	**4.2**	3.4	**4.8**	3.2	
**TG 42:8**	**5.4**	0.8	**6.0**	0.5	**8.4**	5.0	**7.0**	2.9	
**TG 42:9**	**0.2**	0.1	**0.2**	0.1	**0.8**	0.5	**0.5**	0.4	
**TG 44:10**	**5.0**	1.5	**4.1**	1.3	**2.4**	0.8 [a]	**1.2**	0.9 [aa,b]	
**PC**									***m/z***
**PC 30:0+H+**<br>**Cer d44:6+K+**	**0.1**	0.0	**0.2**	0.1	**0.4**	0.1	**0.6**	0.5	706.54
**PC 32:3+H+**<br>**PC 30:0+Na+**	**0.4**	0.1	**0.5**	0.1	**0.8**	0.4	**0.6**	0.5	728.52
**PC 32:1+H+**	**1.0**	0.6	**1.2**	0.8	**1.7**	0.5	**4.3**	1.7 [aa,bb,c]	732.5572
**PC 32:0+H+**	**1.1**	0.6	**2.4**	1.8	**3.0**	0.8	**5.3**	3.1 [a]	734.5723
**PC 34:4+H+**<br>**PC 32:1+Na+**	**4.7**	2.1	**3.6**	1.9	**3.4**	0.9	**3.6**	2.0	754.54
**PC 32:0+Na+**<br>**PC 34:3+H+**	**5.4**	3.2	**8.1**	2.8	**6.7**	1.6	**4.8**	1.2	756.55
**PC 34:1+H+**	**14.2**	6.9	**15.6**	6.9	**14.2**	4.8	**27.0**	3.3 [a,c]	760.5851
**PC 32:1+K+**<br>**PE O-36:4-H+2Na+**<br>**PE O-38:7+Na+**<br>**PE P-36:3-H+2Na+**<br>**PE P-38**	**0.5**	0.2	**0.4**	0.1	**0.5**	0.5	**0.6**	0.4	770.51
**PC 32:0+K+**<br>**PE O-36:3-H+2Na+**<br>**PE O-38:6+Na+**<br>**PE P-36:2-H+2Na+**<br>**PE P-38**	**2.2**	1.4	**1.6**	0.6	**0.7**	0.6	**0.7**	0.5	772.53
**PC 34:2+Na+**<br>**PC 36:5+H+**	**3.9**	1.8	**2.7**	1.1	**9.6**	7.7	**5.2**	2.7	780.56
**PC 34:1+Na+**<br>**PC 36:4+H+**	**39.1**	12.8	**43.5**	7.5	**31.2**	7.4	**30.3**	12.8	782.58
**PC 36:2+H+**	**1.3**	0.6	**1.1**	0.5	**2.7**	0.7	**6.6**	6.9	786.6058
**PC 36:1+H+**	**2.1**	0.5	**2.0**	1.1	**2.1**	0.5 [a]	**1.6**	0.6 [aa]	788.6202
**PC 36:4+Na+**<br>**PC 38:7+H+**	**3.1**	0.9	**3.3**	0.3	**4.6**	2.5	**2.9**	0.7	804.55
**PC 36:2+Na+**<br>**PC 38:5+H+**	**6.3**	2.2	**3.8**	1.0	**6.7**	2.2	**4.5**	1.6	808.59
**PC 36:1+Na+**<br>**PC 38:4+H+**	**12.2**	3.9	**7.9**	2.1	**6.1**	2.6	**3.5**	1.0	810.6

**PC 36:4+K+**<br>**PE O-40:7-H+2Na+**<br>**PE P-40:6-H+2Na+**	**0.8**	0.7	**0.8**	0.0	**0.4**	0.3	**0.5**	0.2	820.53
**PC 38:6+Na+**<br>**PC 40:9+H+**	**1.1**	0.9	**1.5**	0.6	**0.9**	0.2	**0.5**	0.2	828.56
**PC 38:5+Na+**<br>**PC 40:8+H+**	**0.8**	0.2	**0.7**	0.4	**1.2**	0.5	**0.6**	0.2	830.57
**PC 38:4+Na+**	**2.5**	1.8	**2.2**	1.4	**3.6**	2.9	**1.4**	0.5	832.602<br>2
**PC 40:6+Na+**<br>**PC 42:9+H+**	**0.5**	0.6	**0.7**	0.3	**0.4**	0.2	**0.1**	0.1	856.59
**Cer**									
**[Cer_d34:2-H2O+Na]+**<br>**[Cer_m34:1-H2O+Na]+**	**7.4**	4.8	**6.7**	4.9	**38.0**	16.8 aa,bb	**18.9**	2.8	542.49
**[Cer_m36:1-H2O+H]+**	**1.6**	0.6	**2.0**	0.7	**2.4**	0.2 a,b	**1.5**	1.5	548.54
**[Cer_d36:3-H2O+Na]+**<br>**[Cer_d38:6-H2O+H]+**	**8.0**	3.7	**8.5**	2.4	**12.2**	3.2	**6.9**	5.8	568.51
**[Cer_d36:2-H2O+Na]+**<br>**[Cer_d38:5-H2O+H]+**<br>**[Cer_m36:1-H2O+Na]+**	**47.4**	16.0	**59.9**	10.6	**37.0**	12.3	**40.4**	11.7	570.52
**[Cer_d42:3-H2O+Na]+**<br>**[Cer_d44:6-H2O+H]+**	**34.8**	12.7	**19.7**	8.9	**15.9**	7.8	**5.4**	2.0 aa	652.6
**[Cer_d44:6+K]+**<br>**[PC_30:0+H]+**	**1.1**	0.8	**6.1**	4.6	**7.4**	4.5	**27.3**	22.5	706.54

Values are expressed as a percentage of total membrane lipid (mole %) and represent the mean ± SEM (n=4-5). Statistical differences were assessed by ANOVA followed by a Bonferroni post-test. “a” accounts for vs healthy DMSO, “b” for vs healthy TMZ, “c” for tumor DMSO. $^{a, b, c}$ $P < 0.05$; $^{aa, bb, cc}$ $P < 0.01$; $^{aaa, bbb, ccc}$ $P < 0.001$. Abbreviations: PC: phosphatidylcholine; PE: phosphatidylethanolamine; PE P-: phosphatidylethanolamine plasmalogens, PI: phosphatidylinositol; SM: sphingomyelin, DG: diacylglycerol, TG: triacyglycerol, TMZ: temozolomide.

Supplementary Table S2. TCGA-GBM number of genes and identified gene hubs for each of the returned modules generated in the co-expression modular analysis of the TCGA-GBM transcriptome Affy u133a data set.

MODULE	Nº GENES	HUBS
**M1**	300	GPM6A, GPM6B, FEZ1, CTNND2, C1orf61
**M2**	247	FCER1G, CD53, LAPTM5, ITGB2, TLR2
**M3**	129	SYN1, SLC17A7, SLC12A5, GABRA1, RUNDC3A
**M4**	74	COL1A1, COL3A1, COL6A3, COL4A1, COL1A2
**M5**	68	CCNB2, TOP2A, CDC20, BIRC5, UBE2C
**M6**	53	MOG, MBP, MOBP, BCAS1, CAPN3
**Not.Correlated**	319	DDX3Y, EIF1AY, JARID1D, RPS4Y1, NLGN4Y

Supplementary Table S3. TCGA-GBM modules normalized enrichment score and respesctive adjusted p-values (Benjamini-Hochberg) data.

	M1	M2	M3	M4	M5	M6	Not Correlated
**Classical**	2,64	-2,69	-3,53	1,79	-1,09	-3,96	1,10
**Classical Adj P-value**	4,33E-04	2,54E-03	1,38E-03	4,33E-04	2,66E-01	1,03E-03	2,66E-01
**Mesenchymal**	-2,91	3,96	-3,30	3,22	-2,07	-2,51	1,78
**Mesenchymal Adj P-value**	3,12E-04	3,05E-04	3,05E-04	3,05E-04	3,05E-04	3,05E-04	3,05E-04
**Neural**	1,88	1,49	3,38	-2,57	-1,94	2,05	1,37
**Neural Adj P-value**	1,03E-03	1,50E-03	9,98E-04	4,98E-04	4,98E-04	1,03E-03	1,54E-03
**Normal**	1,34	-3,09	4,14	-2,78	-3,22	2,73	-0,92
**Normal Adj P-value**	1,27E-02	2,92E-04	2,92E-04	2,92E-04	2,92E-04	2,92E-04	7,29E-01
**Proneural**	-1,38	-0,39	2,70	-2,94	2,92	3,02	-2,15
**Proneural Adj P-value**	3,53E-03	3,20E-04	3,20E-04	3,20E-04	3,20E-04	3,20E-04	3,20E-04

Supplementary Table S4. Genes contained inside the 6 modules possitivetly correlated with the molecular subtypes (co-expression modular analysis of the TCGA-GBM transcriptome Affy u133a data set).

M1-Classical						M2-Mesenchymal				
ABAT	CLDN5	GJA1	LRIG1	PLA2G5	SPINT2	A2M	CHI3L2	GFPT2	MX1	SKAP2
ABCB1	CLIP2	GLDC	LRP1B	PLEKHB1	SPRY2	ABCC3	CHST2	GIMAP4	MX2	SLA
ABCG2	CLIP3	GLT25D2	LRP4	PLS1	SSPN	ACP5	CLEC2B	GLIPR1	MYLK	SLC16A4
ACSBG1	CLU	GOLSYN	LRRN3	PLSCR4	STEAP1	ADFP	CLEC5A	GPNMB	NNMT	SLC2A5
AGT	CNGA3	GPM6A	LRRTM2	PMAIP1	SUSD4	ADORA3	COLEC12	GPR65	NPL	SLC31A2
ALDH1L1	COBL	GPM6B	LSAMP	PMP2	SV2A	AGTRL1	COPZ2	GYPC	NUPR1	SLPI
ALDOC	CORO2B	GPR177	MAGI2	PMP22	SYT11	AIM1	CP	HAMP	OAS1	SOD2
ANK2	CPE	GPR37	MAP1B	POU3F2	THY1	ALOX5AP	CPVL	HCK	OLFML2B	SPP1
APBA2	CRB1	GPR56	MAP2	PPAP2B	TIMP4	ANGPTL4	CSF1R	HCLS1	OLFML3	SQRDL
APLP1	CREB5	GPRC5A	MAPT	PPP1R3C	TM4SF1	ANXA1	CSTA	HCP5	OLR1	SRGN
APOD	CRIP1	GPX2	MATN2	PRUNE2	TMEM158	ANXA2	CTSC	HLA_DMA	P2RY13	STAB1
APOLD1	CRMP1	GRAMD3	METRN	PSRC1	TMEM47	ANXA4	CTSH	HLA_DMB	PADI2	STEAP3
AQP1	CRYAB	GRIA2	MLC1	PTN	TMOD1	APOBEC3G	CTSS	HLA_DPA1	PBEF1	SYNC1
AQP4	CSPG5	GRIA3	MLLT11	PTPRZ1	TNFRSF21	APOC1	CX3CR1	HLA_DPB1	PDPN	SYNPO
ARC	CSRP2	HEY1	MLPH	RAMP1	TNIK	APOC2	CXCL1	HLA_DQA1	PGDS	tcag7.1314
ARHGEF6	CST3	HNT	MSX1	RASSF2	TPBG	AQP9	CXCL10	HLA_DQB1	PI3	TDO2
ARNT2	CTNNA2	HOPX	MYBPC1	RGS5	TRIB2	ATF3	CXCL14	HLA_DRA	PLA2G2A	TGFBI
ASCL1	CTNND2	HSPA2	NCALD	RHOB	TRIM36	BCL2A1	CXCL2	HLA_DRB1	PLAU	TLR2
ASTN1	CYP46A1	HSPB8	NCAN	RHOBTB3	TRIM9	BIRC3	CXCL3	HMOX1	PLP2	TLR7
ATP1A2	DAAM2	HTR1A	NDP	S100A2	TSPAN7	BLNK	CXCL9	HP	PLTP	TMEM140

ATP1B2	DCLK1	ID3	NDRG2	S100B	TTYH1	BST2	CXCR4	IBSP	PROS1	TMEM176A
ATP6V0E2	DENND2A	ID4	NDRG4	S100P	TUBB2B	C1QA	CYBRD1	IER3	PSMB9	TMEM176B
B3GAT1	DLL3	IMPA2	NELL2	SACS	WASF3	C1QB	DAB2	IFI16	PTGS2	TNFSF10
BAALC	DOK5	ITGA7	NES	SALL1	WFDC2	C1RL	DDR2	IFI30	PTPRC	TREM1
BAI2	DPP6	ITM2A	NFASC	SATB2	WSCD1	C1S	DKFZP586H2123	IFI44	PTX3	TREM2
BAI3	DPYSL3	ITM2C	NKX2_2	SCG2	XYLT1	C21orf62	DPYD	IFITM1	PYCARD	TRIM22
BBOX1	DSP	ITPKB	NLGN1	SCG3	ZBTB16	C21orf7	DSE	IGKC	PYGL	TYROBP
BCAN	DTNA	JAM2	NLGN4X	SCG5	ZBTB20	C3	EBI2	IL10RA	RAB32	UBD
BCHE	EDNRB	JAM3	NMB	SCHIP1	ZEB1	C3AR1	ECM2	IL1B	RARRES1	UPP1
BEX1	EFHD1	KAL1	NME5	SCN1A	ZEB2	C5AR1	EFEMP1	IL1R2	RARRES3	VAMP8
BEX4	EGFL6	KBTBD11	NOVA1	SCRG1	ZFHX4	C8orf4	EVI2A	IL33	RFTN1	VCAM1
BHLHB3	ELOVL2	KCND2	NQO1	SDC1	ZIC1	CAPG	EVI2B	IL6	RGS1	VSIG4
C13orf15	ENO2	KCNIP1	NR2E1	SDC3	ZMAT3	CASP1	F13A1	IL8	RGS2	XAF1
C13orf18	F3	KCNJ16	NR3C2	SEMA5A	ZNF423	CCDC109B	FAM129A	ITGB2	RNASE1	ZFP36
C16orf45	FABP7	KCNMB4	NRCAM	SERPINE2	ZNF91	CCL18	FAS	KCNMB1	RNASE2	ZNF217
C1orf61	FAM107A	KCNN2	NRN1	SEZ6L		CCL2	FBLN5	KCTD12	RNASE4	
C6orf134	FAM131B	KCNN3	NTRK2	SFN		CCL20	FCER1G	LAPTM5	RNASE6	
C9orf61	FAM5B	KHDRBS3	ODZ4	SH3BGR		CCL4	FCGBP	LGALS3	RSAD2	
CA2	FAM5C	KIAA0644	PAQR6	SHROOM2		CD14	FCGR1A	LIF	RTP4	
CAMK2N1	FAM70A	KIF1B	PAX6	SLC1A2		CD163	FCGR2A	LOX	S100A10	
CAP2	FEZ1	KLF5	PCAF	SLC1A3		CD302	FCGR2B	LTF	S100A11	
CCND2	FGF14	KLHDC8A	PCDH17	SLC22A17		CD44	FER1L3	LY75	S100A4	

CD55	FHL1	KLHL4	PCDH8	SLC24A3		CD48	FLJ20273	LY86	S100A8	
CDH10	FJX1	KRT18	PCDH9	SLC4A4		CD52	FLJ22662	LY96	S100A9	
CDH4	FLJ10781	LAMP3	PCSK1N	SLC6A1		CD53	FOLR2	LYVE1	SAMSN1	
CDO1	FOXG1	LGI1	PDE4B	SLCO1C1		CD69	FOS	LYZ	SDC2	
CENTD1	FXYD6	LHFP	PDE8B	SLITRK3		CD74	FXYD5	MAFB	SELL	
CES1	FYN	LHX2	PDGFA	SMARCD3		CEBPA	FZD7	MAN1C1	SEPP1	
ChGn	FZD3	LMO2	PDZD2	SNCAIP		CECR1	G0S2	MAOB	SERPINA1	
CHL1	GABRB1	LOC57228	PERP	SOBP		CFD	GALNAC4S_6ST	MGP	SERPINA3	
CHN1	GALNT3	LPHN3	PHLPP	SOX9		CFI	GBP1	MNDA	SERPINB1	
CITED1	GAP43	LPL	PID1	SPARC		CH25H	GBP2	MS4A4A	SERPINF1	
CKB	GATM	LPPR4	PIPOX	SPARCL1		CHI3L1	GEM	MS4A6A	SERPING1	

**M3-Neural-Normal**			**M4-Mesenchymal**		**M5-Proneural**		**M6-Proneural-Normal**
ATP1B1	PGBD5	GABRA2	ACTA2	PDIA5	APOBEC3B	SOX11	AMOTL2
BASP1	PRKCB1	HPCA	ACTG2	PDLIM1	ASPM	SOX4	ASPA
RTN1	RUNDC3A	DGKB	ACTN1	POSTN	BIRC5	SPC25	ATP10B
KIF5C	NAP1L2	CHGA	ADAMTS1	PRSS23	BLM	STK32B	BCAS1
C14orf132	DBC1	NEFM	AEBP1	PXDN	BUB1B	THSD7A	C20orf39
STXBP1	C1orf115	FXYD7	ANGPT2	RCAN1	CCNA2	TMEM45A	C20orf42
ENC1	PEG3	KIAA1107	ASPN	SERPINE1	CCNB1	TMSL8	CA10
PKIA	SYT1	AK5	CA12	SERPINH1	CCNB2	TOP2A	CAPN3
C10orf38	SH3GL2	TAC1	CD93	SLC2A10	CDC2	TOX3	CNTN1
OMG	NPTX1	GABRA1	CDH11	SNAI2	CDC20	TPX2	DNM3
TCEAL2	PAK3	PPP1R1A	CNN3	SPON2	CDC7	TRIP13	ELMO1
NUDT11	CRYM	NELL1	COL15A1	SPRY1	CDK4	TTK	ENPP2
PSD3	FGF13	LY6H	COL1A1	SRPX2	CDKN3	TYMS	EPB41L3
NAP1L3	NPY	MYT1L	COL1A2	TAGLN	CENPF	UBE2C	EPHB1
KIF1A	ERC2	FGF9	COL3A1	TGFB1I1	CHD7	ZWINT	ERBB3
REEP1	GNG3	PNOC	COL4A1	TIMP1	CHIC2		FA2H
CYFIP2	CAMTA1	WIF1	COL4A2	TNC	CKS1B		FGF12
SNAP25	NRXN2	NEFH	COL5A1	TRIP6	CKS2		GNAI1
MAPK10	HSPA12A	SNCB	COL5A2	VEGFA	CLGN		GPR17
INPP5F	GNAO1	ARPP_21	COL6A2	VWF	DCX		GRM3
TAGLN3	SCN3B	ACTL6B	COL6A3	ZYX	DEPDC1		HRASLS
EEF1A2	NEFL	SLC17A6	CRISPLD2		DLG7		KIAA1598
RCAN2	HMP19	PDYN	CTGF		DTL		KIF21B

GPRASP1	NRIP3		CTSK		ECT2		KLK6
GABBR1	TMEM35		CXCL12		EZH2		KLRC3
ATP6V1G2	CAMK2B		CYP1B1		FAM60A		MAGEH1
PRKAR2B	KCNK1		CYR61		FAM64A		MAL
SERPINI1	GABBR2		DCN		FANCI		MBP
DNAJC12	CCK		EDNRA		GINS1		MOBP
GUCY1B3	SNAP91		EFEMP2		GINS2		MOG
OLFM1	MYRIP		ELTD1		GNG4		MYOT
STMN4	INA		EMP1		HIST1H1C		OPCML
STMN2	RAPGEF4		EMP3		HMMR		PLLP
ELAVL4	SYNGR3		F2R		IGF2BP3		PLP1
NBEA	RGS4		FAP		INSM1		PPP1R16B
DYNC1I1	SLC17A7		FILIP1L		KIF11		PTGDS
AMPH	SYN1		FSTL1		KIF15		RAB33A
NAV3	SLITRK5		HRH1		KIF20A		RAPGEF5
C20orf103	NRXN1		IGFBP2		KIF4A		RNF144A
AGXT2L1	HS3ST2		IGFBP4		MAD2L1		RP11_35N6.1
DIRAS2	SST		LAMB1		MCM2		S100A1
KIAA0513	CHGB		LOXL1		MELK		SATB1
RAP1GAP	DDX25		LTBP2		MLF1IP		SCN3A
NRGN	TAC3		LUM		MYBL1		SOX10
ZNF365	PIP3_E		MFAP2		NDC80		SPOCK1
CA11	RALYL		MMP2		NMU		ST18
VSNL1	SLC12A5		MMP9		NUP107		TF
EHD3	NMNAT2		MXRA5		NUSAP1		TMEFF1
NOL4	RASL10A		MYL9		OIP5		TUBB4
PHACTR1	SV2B		NID1		PBK		UGT8
CHST1	HPCAL4		NID2		PRC1		WASF1
SNCA	KCNB1		P4HA2		PTTG1		ZNF536
PTPRD	NTSR2		PCOLCE		RRM2		ZNF804A

Supplementary Table S5. TCGA-GBM modules gene ontology (GO). Top ten annotations for GO biological processes (BP) filtered by FDR (≤ 0.05).

	Category	Term	%	PValue	Genes	Fold Enrichment	FDR
M1	GOTERM_BP_DIRECT	GO:0007399~nervous system development	9,56	2,98E-12	SEMA5A, NLGN1, NRN1, BEX1, CRMP1, PTN, NR2E1, CYP46A1, EDNRB, NDP, ZNF423, GPM6B, LGI1, APLP1, LSAMP, DCLK1, ZEB2, FGF14, DOK5, MAP1B, FEZ1, FABP7, CSPG5, APBA2, SCRG1, ITM2A	5,83	4,95E-09
	GOTERM_BP_DIRECT	GO:0001764~neuron migration	5,51	8,40E-10	GPM6A, NTRK2, FZD3, SATB2, PAX6, ASCL1, DCLK1, GJA1, TUBB2B, CHL1, NRCAM, FYN, MAPT, ASTN1, MATN2	9,19	6,97E-07
	GOTERM_BP_DIRECT	GO:0007417~central nervous system development	5,15	4,38E-08	ARNT2, NCAN, ZBTB16, PAX6, S100B, DCLK1, BCAN, ZEB1, PTPRZ1, ID3, NRCAM, FYN, NES, TIMP4	7,51	2,42E-05
	GOTERM_BP_DIRECT	GO:0007155~cell adhesion	8,09	1,01E-05	SEMA5A, NLGN1, MYBPC1, EGFL6, NLGN4X, NCAN, TPBG, CTNND2, APLP1, LSAMP, ATP1B2, THY1, PCDH17, RHOB, BCAN, CDH4, PDZD2, CHL1, FEZ1, SSPN, ITGA7, CTNNA2	3,08	4,18E-03
	GOTERM_BP_DIRECT	GO:0007612~learning	2,94	2,69E-05	CLDN5, NTRK2, BCHE, ARC, NLGN4X, FYN, PTN, SLC6A1	9,03	7,44E-03
	GOTERM_BP_DIRECT	GO:0060291~long-term synaptic potentiation	2,57	2,37E-05	NTRK2, NLGN1, SERPINE2, LRRTM2, PTN, NR2E1, S100B	11,85	7,44E-03
	GOTERM_BP_DIRECT	GO:0030203~glycosaminoglycan metabolic process	2,21	7,50E-05	BCAN, NCAN, SDC3, B3GAT1, CSPG5, SDC1	13,31	1,78E-02
	GOTERM_BP_DIRECT	GO:0050808~synapse organization	2,21	1,43E-04	NLGN1, NFASC, NLGN4X, CTNND2, LRRTM2, LRP4	11,70	2,96E-02
	GOTERM_BP_DIRECT	GO:0016337~single organismal cell-cell adhesion	3,31	1,73E-04	DSP, TTYH1, CDH10, CTNND2, NRCAM, SOX9, CTNNA2, THY1, JAM2	5,73	3,20E-02

	Category	Term	%	PValue	Genes	Fold Enrichment	FDR
M2	GOTERM_BP_DIRECT	GO:0006954~inflammatory response	18,83	1,19E-26	SERPINA3, CSF1R, CXCL9, C5AR1, ITGB2, LY75, LY96, CXCR4, CXCL1, CXCL3, PTGS2, CXCL2, C3, PYCARD, IFI16, CCL4, STAB1, BLNK, C3AR1, SPP1, CCL2, OLR1, CD14, CCL18, ANXA1, CCL20, LY86, FOS, LYZ, CXCL10, HCK, IL6, IL1B, CHI3L1, FAS, TLR7, PTX3, FOLR2, S100A9, S100A8, CHST2, TLR2	8,74	1,65E-23
	GOTERM_BP_DIRECT	GO:0006955~immune response	15,70	3,86E-18	CXCL9, GPR65, C5AR1, AQP9, LY75, CXCL1, CXCL3, CXCL14, CXCL2, CTSS, C3, IGKC, RGS1, CCL4, TNFSF10, CCL2, GBP2, CCL18, FCGR1A, TRIM22, CTSC, CD74, CCL20, IL1R2, LIF, GEM, CXCL10, IL6, OAS1, SLPI, IL1B, FAS, FCGR2B, HAMP, TLR2	6,55	2,67E-15
	GOTERM_BP_DIRECT	GO:0045087~innate immune response	13,90	2,46E-14	C1QB, COLEC12, C1QA, CSF1R, C1S, CFI, LY96, TREM2, TREM1, PYCARD, LGALS3, IFI16, IGKC, C1RL, CLEC5A, CD14, FCER1G, ANXA1, APOBEC3G, MX2, MX1, LY86, BST2, TYROBP, SLPI, SERPING1, TLR7, PTX3, S100A9, S100A8, TLR2	5,68	1,13E-11
	GOTERM_BP_DIRECT	GO:0032496~response to lipopolysaccharide	8,52	2,78E-12	CXCL9, VCAM1, MAOB, IL10RA, C5AR1, LY96, CXCL1, FOS, CXCL3, PTGS2, SOD2, CXCL2, CXCL10, SLPI, CASP1, FAS, ACP5, S100A8, TLR2	9,13	9,62E-10
	GOTERM_BP_DIRECT	GO:0030593~neutrophil chemotaxis	5,83	3,25E-11	FCER1G, CCL20, ITGB2, C5AR1, CXCL3, TREM1, LGALS3, IL1B, CCL4, CCL2, CCL18, S100A9, S100A8	15,53	8,98E-09
	GOTERM_BP_DIRECT	GO:0051607~defense response to virus	7,62	3,07E-10	IL33, IFITM1, CXCL9, APOBEC3G, RSAD2, MX2, MX1, BST2, PYCARD, CXCL10, IL6, PTPRC, IFI16, OAS1, TLR7, GBP1, TRIM22	8,12	7,07E-08
	GOTERM_BP_DIRECT	GO:0006935~chemotaxis	6,73	4,38E-10	CX3CR1, CXCL9, CCL20, C5AR1, CXCR4, CXCL1, RNASE2, CXCL2, CXCL14, CXCL10, PLAU, C3AR1, CCL2, PLP2, CCL18	9,69	8,65E-08
	GOTERM_BP_DIRECT	GO:0007165~signal transduction	17,94	1,63E-08	CSF1R, CXCL9, C5AR1, LY75, CXCL1, CXCL14, C3, PYCARD, PLAU, RGS1, PDPN, CCL4, CLEC5A, CASP1, TNFSF10, CCL2, CCL18, FCGR1A, S100A11, CD53, CD74, ANXA1, CCL20, ANXA4, MX1, LYVE1, GEM, CXCL10, TYROBP, EVI2A, IL1B, ZNF217, FAS, CD48, CD69, FCGR2B, S100A9, SKAP2, DDR2, TLR2	2,72	2,81E-06
	GOTERM_BP_DIRECT	GO:0070098~chemokine-mediated signaling pathway	4,93	1,96E-08	CX3CR1, CXCL10, CXCL9, CCL20, CCL4, CCL2, CXCR4, CXCL1, CXCL3, CCL18, CXCL2	12,21	3,02E-06
	GOTERM_BP_DIRECT	GO:0045071~negative regulation of viral genome replication	4,04	3,07E-08	BST2, IFITM1, IFI16, RSAD2, APOBEC3G, OAS1, SLPI, MX1, LTF	17,74	4,25E-06

	Category	Term	%	PValue	Genes	Fold Enrichment	FDR
M3	GOTERM_BP_DIRECT	GO:0007268~chemical synaptic transmission	14,88	1,82E-13	GABBR2, SNAP25, SLC12A5, SYT1, NRXN1, NRXN2, PDYN, SNAP91, SYN1, PTPRD, SST, NPY, PNOC, SLITRK5, AMPH, NPTX1, TAC1, SNCB	11,66	1,18E-10
	GOTERM_BP_DIRECT	GO:0007269~neurotransmitter secretion	4,96	1,78E-05	SNAP25, SYT1, NRXN1, STXBP1, NRXN2, SYN1	18,29	5,76E-03
	GOTERM_BP_DIRECT	GO:0033693~neurofilament bundle assembly	2,48	1,20E-04	NEFL, NEFM, NEFH	155,48	2,60E-02
	GOTERM_BP_DIRECT	GO:0007214~gamma-aminobutyric acid signaling pathway	3,31	3,55E-04	GABRA2, GABBR2, GABRA1, GABBR1	28,27	5,13E-02
	GOTERM_BP_DIRECT	GO:0007218~neuropeptide signaling pathway	4,96	4,66E-04	NPY, PNOC, TAC3, PDYN, TAC1, NTSR2	9,24	5,13E-02
	GOTERM_BP_DIRECT	GO:0007612~learning	4,13	4,75E-04	SLC12A5, PRKAR2B, NRXN1, ELAVL4, FGF13	13,64	5,13E-02
	GOTERM_BP_DIRECT	GO:0014047~glutamate secretion	3,31	7,34E-04	SNAP25, SYT1, STXBP1, SLC17A7	22,21	6,79E-02
	GOTERM_BP_DIRECT	GO:0061564~axon development	2,48	1,75E-03	NEFL, NEFM, NEFH	46,64	1,42E-01
	GOTERM_BP_DIRECT	GO:0007399~nervous system development	6,61	2,41E-03	OLFM1, NELL1, MYT1L, ENC1, FGF13, SCN3B, LY6H, NRGN	4,33	1,74E-01
	GOTERM_BP_DIRECT	GO:0007600~sensory perception	2,48	3,00E-03	PNOC, PDYN, NTSR2	35,88	1,94E-01

	Category	Term	%	PValue	Genes	Fold Enrichment	FDR
M4	GOTERM_BP_DIRECT	GO:0030198~extracellular matrix organization	31,51	3,22E-26	POSTN, VWF, LUM, SERPINE1, TNC, LAMB1, NID1, NID2, DCN, CYR61, LOXL1, COL1A1, COL3A1, COL1A2, COL4A2, COL5A1, COL4A1, CRISPLD2, COL6A2, COL5A2, MFAP2, PXDN, COL6A3	28,15	1,85E-23
	GOTERM_BP_DIRECT	GO:0030574~collagen catabolic process	17,81	2,32E-17	COL15A1, MMP2, MMP9, COL1A1, COL3A1, COL1A2, COL4A2, COL5A1, COL4A1, CTSK, COL6A2, COL5A2, COL6A3	48,73	6,66E-15
	GOTERM_BP_DIRECT	GO:0007155~cell adhesion	26,03	2,49E-13	SPON2, POSTN, COL15A1, VWF, TGFB1I1, TNC, LAMB1, NID2, CYR61, CTGF, COL1A1, CXCL12, COL5A1, FAP, COL6A2, CDH11, ZYX, CYP1B1, COL6A3	9,93	4,76E-11
	GOTERM_BP_DIRECT	GO:0030199~collagen fibril organization	10,96	2,01E-10	COL1A1, COL3A1, COL1A2, COL5A1, LUM, COL5A2, SERPINH1, CYP1B1	49,21	2,88E-08
	GOTERM_BP_DIRECT	GO:0009612~response to mechanical stimulus	10,96	4,17E-09	RCAN1, COL3A1, POSTN, CXCL12, ANGPT2, IGFBP2, TNC, DCN	32,53	4,79E-07
	GOTERM_BP_DIRECT	GO:0001501~skeletal system development	12,33	8,79E-08	COL1A1, COL3A1, POSTN, COL1A2, IGFBP4, CDH11, COL5A2, AEBP1, MMP9	15,76	8,41E-06
	GOTERM_BP_DIRECT	GO:0001525~angiogenesis	13,70	3,10E-07	COL15A1, SRPX2, ANGPT2, COL4A2, FAP, MMP2, SERPINE1, CYP1B1, CTGF, VEGFA	10,76	2,55E-05
	GOTERM_BP_DIRECT	GO:0071230~cellular response to amino acid stimulus	8,22	1,36E-06	COL1A1, COL3A1, COL1A2, COL4A1, MMP2, COL5A2	30,62	9,73E-05
	GOTERM_BP_DIRECT	GO:0022617~extracellular matrix disassembly	8,22	1,49E-05	MMP2, CTSK, TIMP1, NID1, MMP9, DCN	18,94	9,50E-04
	GOTERM_BP_DIRECT	GO:0001666~response to hypoxia	9,59	7,44E-05	PDLIM1, EDNRA, POSTN, CXCL12, ANGPT2, MMP2, VEGFA	9,76	4,27E-03

	Category	Term	%	PValue	Genes	Fold Enrichment	FDR
M5	GOTERM_BP_DIRECT	GO:0051301~cell division	32,81	5,56E-19	UBE2C, BUB1B, CDC7, KIF11, NDC80, ZWINT, CKS1B, CCNA2, CDC20, CCNB2, TPX2, CENPF, CCNB1, PTTG1, CDK4, CKS2, BIRC5, OIP5, FAM64A, MAD2L1, SPC25	15,99	2,65E-16
	GOTERM_BP_DIRECT	GO:0007067~mitotic nuclear division	25,00	1,14E-14	BUB1B, KIF11, NDC80, KIF15, CCNA2, CDC20, ASPM, CCNB2, TPX2, CENPF, PTTG1, PBK, BIRC5, OIP5, FAM64A, SPC25	17,20	2,70E-12
	GOTERM_BP_DIRECT	GO:0007062~sister chromatid cohesion	14,06	3,91E-09	CDC20, CENPF, NUP107, BIRC5, BUB1B, NDC80, ZWINT, MAD2L1, SPC25	23,29	6,21E-07
	GOTERM_BP_DIRECT	GO:0007052~mitotic spindle organization	7,81	4,30E-06	CCNB1, TTK, KIF11, NDC80, SPC25	44,42	4,83E-04
	GOTERM_BP_DIRECT	GO:0007059~chromosome segregation	9,38	5,08E-06	TOP2A, CENPF, OIP5, KIF11, NDC80, SPC25	23,52	4,83E-04
	GOTERM_BP_DIRECT	GO:0031145~anaphase-promoting complex-dependent catabolic process	9,38	1,06E-05	CDC20, CCNB1, PTTG1, UBE2C, BUB1B, MAD2L1	20,24	8,44E-04
	GOTERM_BP_DIRECT	GO:0000082~G1/S transition of mitotic cell cycle	9,38	3,68E-05	RRM2, CDK4, CDC7, TYMS, CDKN3, MCM2	15,68	2,51E-03
	GOTERM_BP_DIRECT	GO:0007094~mitotic spindle assembly checkpoint	6,25	5,22E-05	CENPF, BUB1B, TTK, MAD2L1	53,31	3,11E-03
	GOTERM_BP_DIRECT	GO:0000070~mitotic sister chromatid segregation	6,25	1,04E-04	NUSAP1, NDC80, ZWINT, MAD2L1	42,65	5,50E-03
	GOTERM_BP_DIRECT	GO:0051436~negative regulation of ubiquitin-protein ligase activity involved in mitotic cell cycle	7,81	1,36E-04	CDC20, CCNB1, UBE2C, BUB1B, MAD2L1	18,77	6,47E-03

	Category	Term	%	PValue	Genes	Fold Enrichment	FDR
M6	GOTERM_BP_DIRECT	GO:0042552~myelination	8,33	2,52E-04	PLLP, MAL, MBP, KLK6	31,74	4,35E-02
	GOTERM_BP_DIRECT	GO:0007417~central nervous system development	10,42	2,95E-04	UGT8, MOG, MAL, MBP, KLK6	15,21	4,35E-02
	GOTERM_BP_DIRECT	GO:0007422~peripheral nervous system development	6,25	1,87E-03	UGT8, ERBB3, SOX10	45,63	1,84E-01
	GOTERM_BP_DIRECT	GO:0021762~substantia nigra development	6,25	7,62E-03	S100A1, PLP1, MBP	22,35	5,62E-01
	GOTERM_BP_DIRECT	GO:0002175~protein localization to paranode region of axon	4,17	1,07E-02	UGT8, EPB41L3	182,52	6,30E-01
	GOTERM_BP_DIRECT	GO:0008366~axon ensheathment	4,17	1,86E-02	PLP1, MBP	104,30	7,30E-01
	GOTERM_BP_DIRECT	GO:0030913~paranodal junction assembly	4,17	1,86E-02	UGT8, EPB41L3	104,30	7,30E-01
	GOTERM_BP_DIRECT	GO:0001766~membrane raft polarization	4,17	2,12E-02	PLLP, MAL	91,26	7,30E-01
	GOTERM_BP_DIRECT	GO:0006928~movement of cell or subcellular component	6,25	2,23E-02	ENPP2, ELMO1, WASF1	12,73	7,30E-01
	GOTERM_BP_DIRECT	GO:0007268~chemical synaptic transmission	8,33	2,63E-02	GRM3, PLP1, MBP, FGF12	6,08	7,77E-01

Supplementary Table S6. Top ten GO enrichment for biological processes of PLA2G5, FABP7, ELOVL2, PLA2G2A, and ALOX5AP genes.

#term ID	term description	observed gene count	background gene count	strength	false discovery rate	Labels
GO:1901570	fatty acid derivative biosynthetic process	3	82	2,16	0,00024	ELOVL2,PLA2G5,ALOX5AP
GO:0036149	phosphatidylinositol acyl-chain remodeling	2	16	2,69	0,0008	PLA2G5,PLA2G2A
GO:0019370	leukotriene biosynthetic process	2	18	2,64	0,0008	PLA2G5,ALOX5AP
GO:0036148	phosphatidylglycerol acyl-chain remodeling	2	18	2,64	0,0008	PLA2G5,PLA2G2A
GO:0036150	phosphatidylserine acyl-chain remodeling	2	21	2,57	0,0008	PLA2G5,PLA2G2A
GO:0050482	arachidonic acid secretion	2	24	2,51	0,0008	PLA2G5,PLA2G2A
GO:0036152	phosphatidylethanolamine acyl-chain remodelii	2	26	2,48	0,0008	PLA2G5,PLA2G2A
GO:0036151	phosphatidylcholine acyl-chain remodeling	2	27	2,46	0,0008	PLA2G5,PLA2G2A
GO:0006654	phosphatidic acid biosynthetic process	2	38	2,31	0,0008	PLA2G5,PLA2G2A
GO:0016042	lipid catabolic process	3	265	1,65	0,0008	FABP7,PLA2G5,PLA2G2A

Supplementary Figure S1. Saturated, MUFA, DUFA, PUFA composition of healthy and tumor tissue.

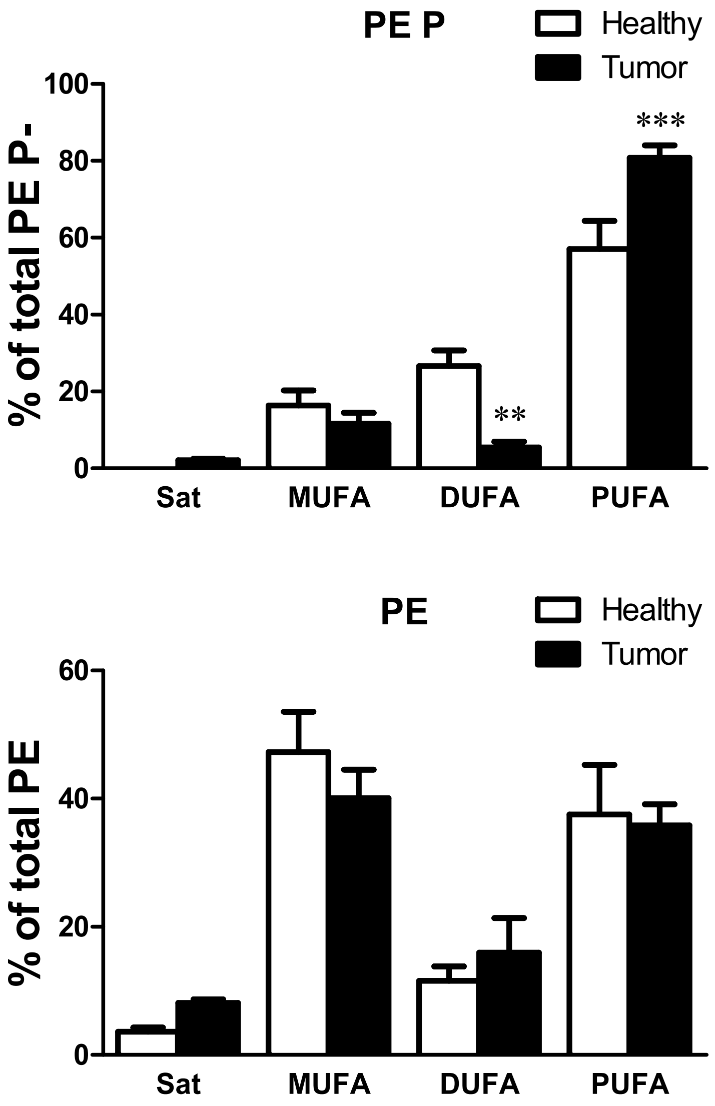

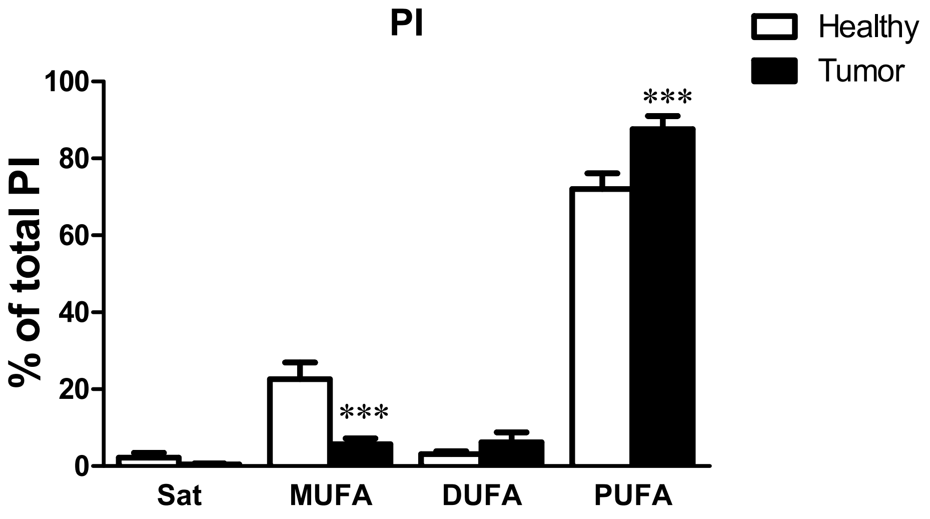

Tumor DMSO vs Tz

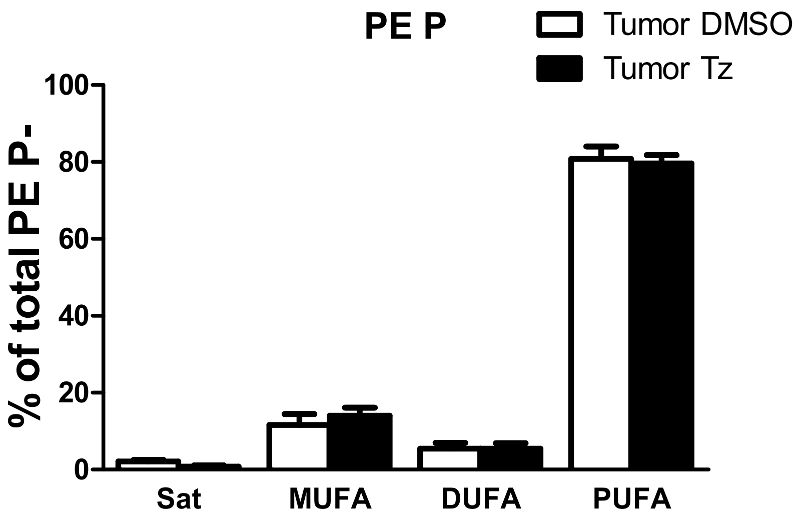

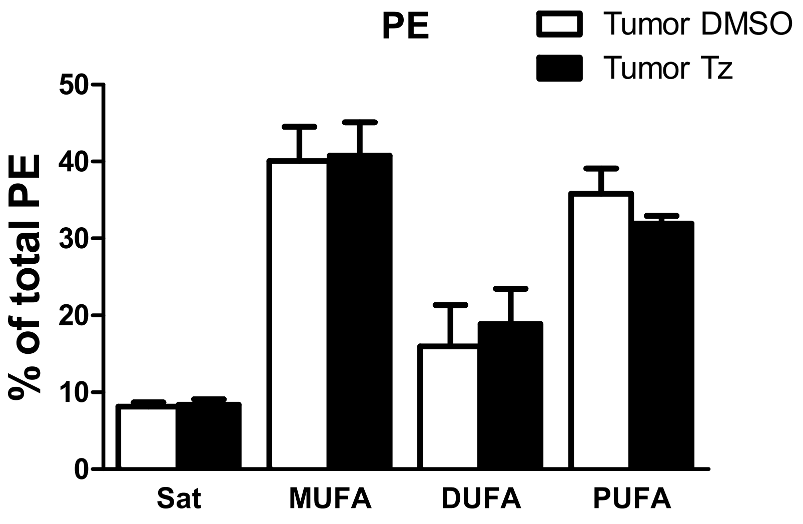

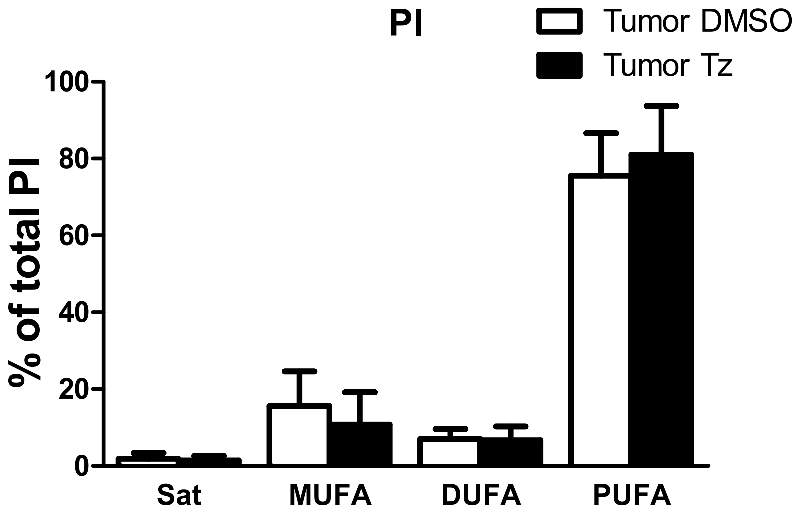

Healthy DMSO vs Tz

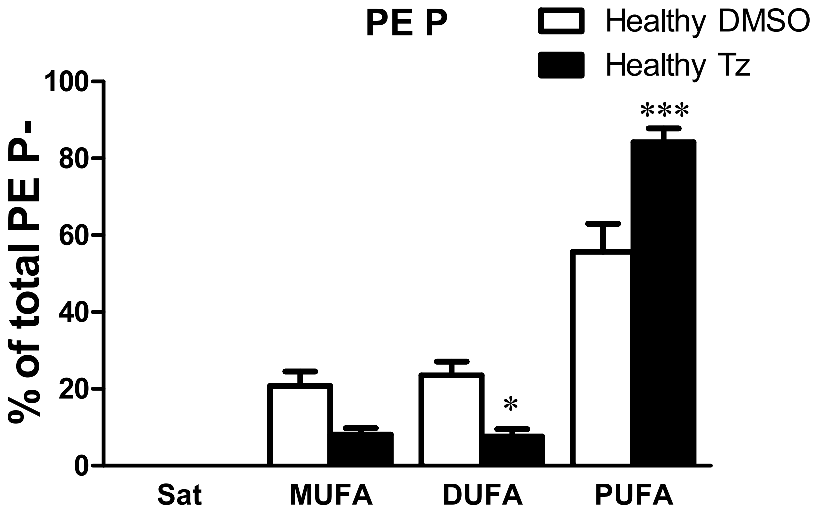

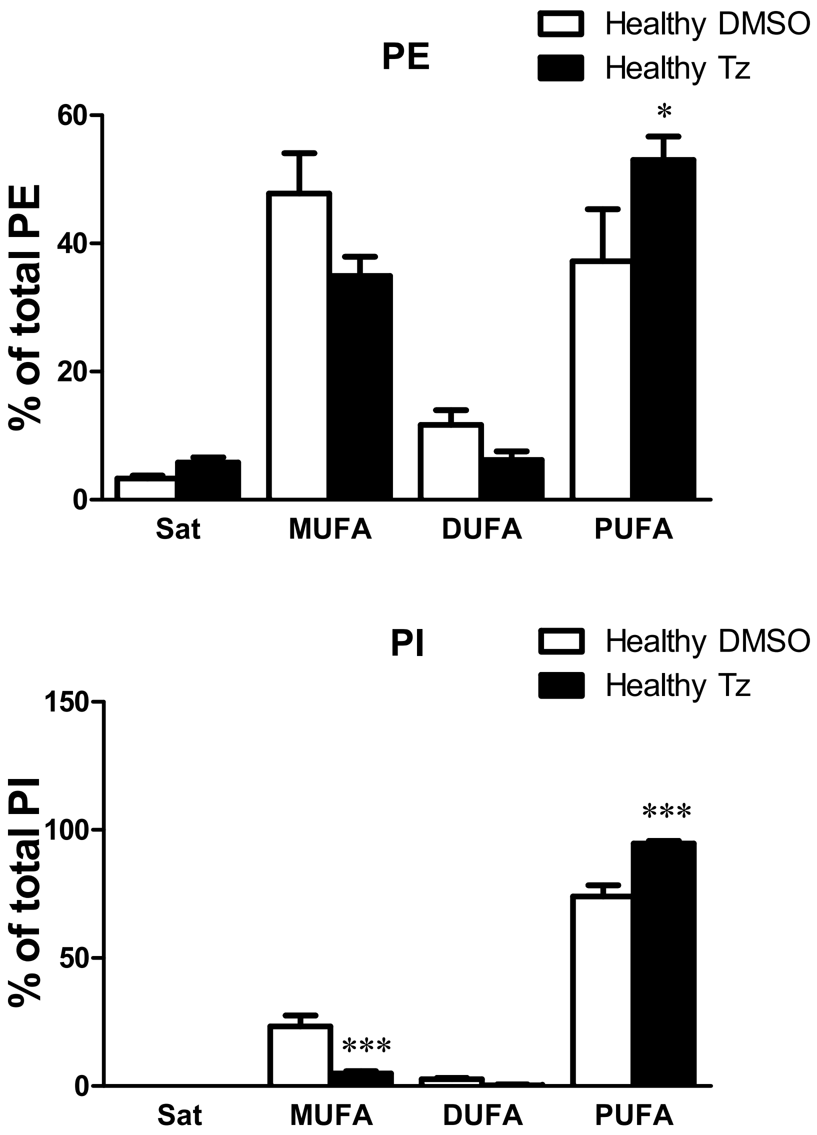
PE
Healthy DMSO
Healthy Tz
% of total PE
60
40
20
0
*
Sat
MUFA
DUFA
PUFA
PI
Healthy DMSO
Healthy Tz
% of total PI
150
100
50
0
***
***
Sat
MUFA
DUFA
PUFA

Supplementary Figure S2. Significantly associated disease-specific survival analysis of the lipid-related genes identified in the molecular subtypes correlated M1-Classical, M2-mesenchymal, and M6-Proneural. **a.** PLA2G5, **b.** FABP7, **c.** ALOX5AP, **d.** ELOVL2, **e.** PLA2G2A, **f.** UGT8, **g.** ALOX15B.

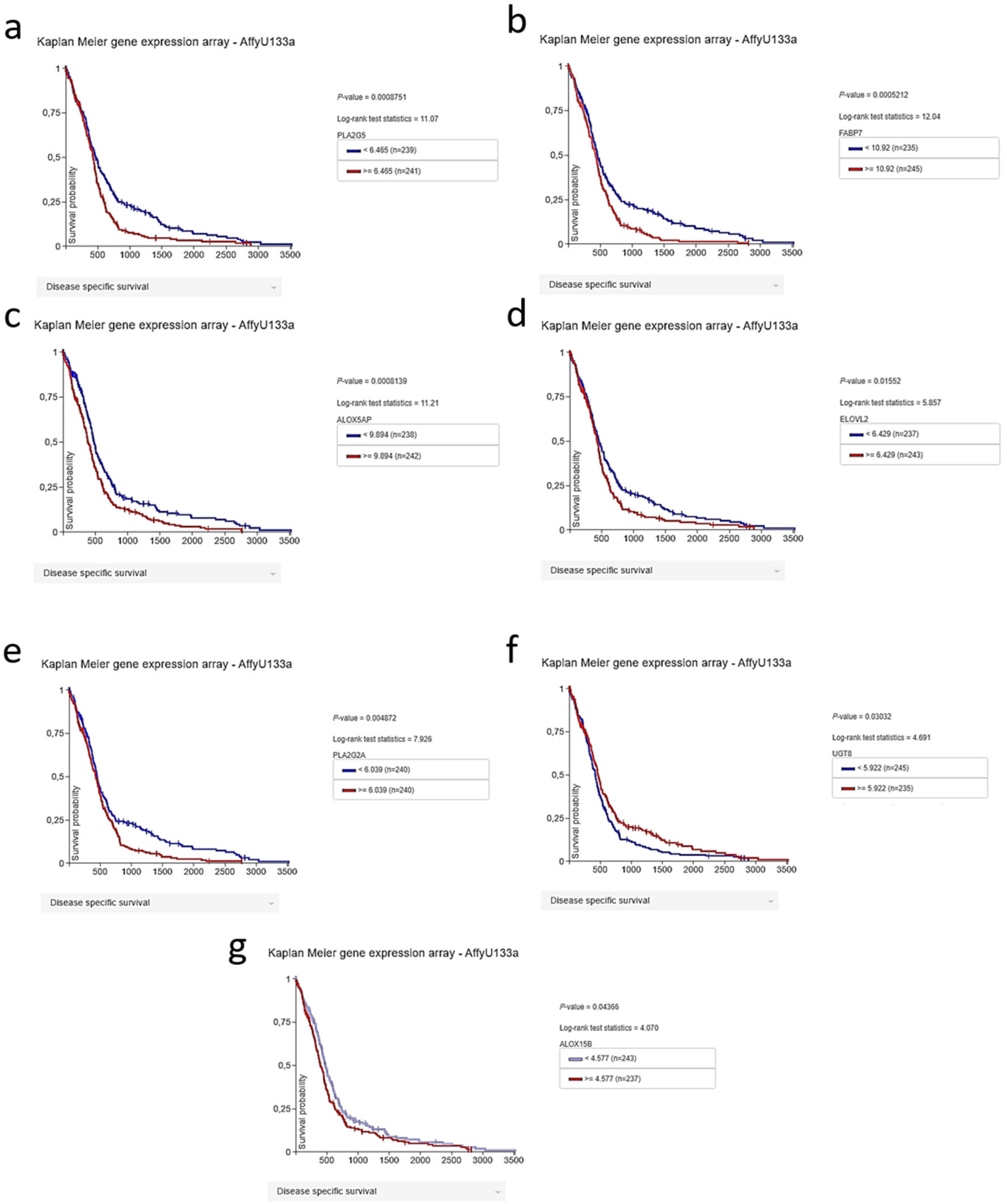

Supplement: Supplementary file 1 [file ijms-23-02949-s001.zip › ijms-1605617-supplementary.pdf]
